# Cross-Reactive Immunity Among Flaviviruses

**DOI:** 10.3389/fimmu.2020.00334

**Published:** 2020-02-26

**Authors:** Abhay P. S. Rathore, Ashley L. St. John

**Affiliations:** ^1^Department of Pathology, Duke University Medical Center, Durham, NC, United States; ^2^Program in Emerging Infectious Diseases, Duke-National University of Singapore Medical School, Singapore, Singapore; ^3^Department of Microbiology and Immunology, Yong Loo Lin School of Medicine, National University of Singapore, Singapore, Singapore; ^4^SingHealth Duke-National University of Singapore Global Health Institute, Singapore, Singapore

**Keywords:** flavivirus, dengue, Zika, yellow fever, tick-borne encephalitis, cross-protection, vector-borne

## Abstract

Flaviviruses consist of significant human pathogens responsible for hundreds of millions of infections each year. Their antigenic relationships generate immune responses that are cross-reactive to multiple flaviviruses and their widespread and overlapping geographical distributions, coupled with increases in vaccination coverage, increase the likelihood of exposure to multiple flaviviruses. Depending on the antigenic properties of the viruses to which a person is exposed, flavivirus cross-reactivity can be beneficial or could promote immune pathologies. In this review we describe our knowledge of the functional immune outcomes that arise from varied flaviviral immune statuses. The cross-reactive antibody and T cell immune responses that are protective versus pathological are also addressed.

## Introduction to Flaviviruses

**FIGURE 1:**
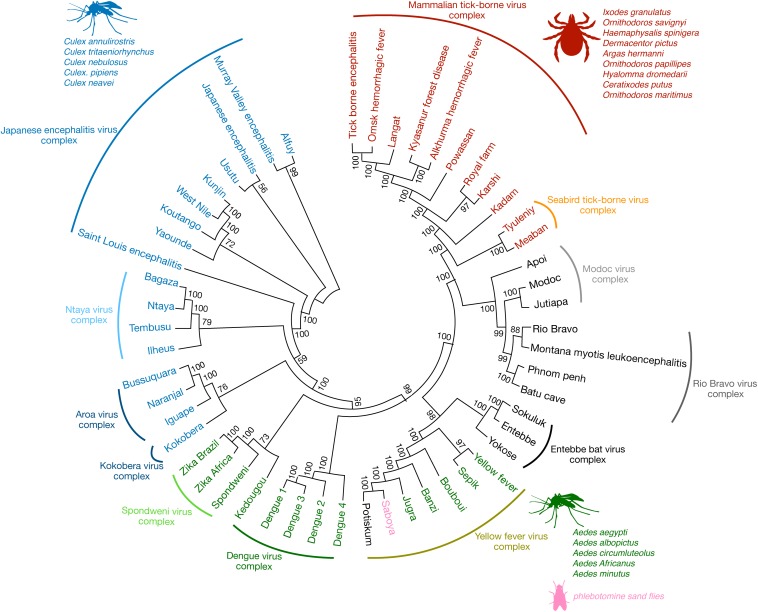
The antigenic relationships among flaviviruses. Phylogenetic analysis demonstrates that flaviviruses cluster not just antigenically but also group according to their known transmission vectors. Some of the most significant flaviviral human pathogens belong to the JEV, Spondweni, DENV, YFV, and mammalian TBV serocomplexes, respectively (arched lines cover viruses of the same serocomplex). Some of their most common vectors are also listed, such as the mosquito species *Culex* (blue) and *Aedes* (green) and various species of ticks (red). Other flaviviruses have no known vector, for example, viruses of the Modoc, Rio Bravo, and Entebbe bat virus complex (black). Among the *Aedes* mosquito-borne viruses of the YFV serocomplex, Saboya virus (pink) has been successfully isolated from the phlebotomine sand flies (85). Phylogenetic analysis was conducted using molecular evolutionary genetic analysis (MEGA-7) software (86). The full-length polyprotein amino acid sequences from various flaviviruses were obtained from the NCBI database and pairwise aligned using Clustal W. The phylogenetic tree was constructed by using the maximum likelihood method based on the Jones-Taylor-Thornton (JTT) matrix-based model (87). The consensus tree representing 200 bootstrap is presented (88). Branches that were reproduced in less than 50% bootstrap replicates are collapsed. The nodes show bootstrap support values from replicates.

Flaviviruses are enveloped single-stranded positive-sense RNA viruses that share conserved structural and genomic features (1). The viral genome encodes for three structural and seven non-structural proteins that are needed for virus replication and assembly (1). Some of the most prominent mosquito-borne flaviviral human pathogens include the hemorrhagic fever viruses, dengue (DENV) and yellow fever (YFV), and neurotropic viruses, such as West Nile (WNV), Japanese encephalitis (JEV), Saint Louis encephalitis (SLEV), and Zika (ZIKV). Yet other flaviviruses that are human pathogens, such as Kyasanur forest disease (hemorrhagic) and Powassan (encephalitic) viruses, are tick-borne. Additional flaviviruses have no known vector (2) while others are thought to be restricted to insects or bats and are not reported to cause human disease (3). Phylogenetic analysis has shown that flaviviruses cluster in genomic similarity according to their dominant vector (Figure 1), which also is a major contributing factor to the often-overlapping global distribution patterns of each flavivirus. The genetic differences amongst flaviviruses result in both conserved and species-specific attributes, such as cellular and tissue tropism upon infection and, importantly for the purposes of this review, antigenic properties.

## Classification and Antigenic Relationships Among Flaviviruses

The name flavivirus (flavus- means “yellow” in Latin) stems from early research done on the YFV vaccine in 1930s, for which a Nobel Prize was awarded to Marx Theiler in 1951 (4). In the initial classification scheme, arthropod-borne viruses were classified based on their ability to replicate and transmit through arthropods and distributed in to two groups belonging to the family *Togaviridae* (5). Group A comprised of arthropod-borne viruses such as chikungunya and sindbis (now in the genus alphavirus) and Group B comprised of viruses such as YFV and DENV (now in the genus flavivirus, and the subjects of this review). Because of the distinct antigenic characteristics of flaviviruses, they were later classified in to the new genus, flavivirus of the family *Flaviviridae* (6). The first arthropod-borne virus cross-reactivity was observed in complement fixation tests (7), which allows a complement reaction to occur on the surface of red blood cells (RBCs) when serum is added in the presence of a known antigen. Later, the hemaggIutination inhibition assay, involving inhibition of virus-induced hemagglutination (or aggregation of RBCs) in the presence of serum was used to describe flavivirus cross-reactivity (8). Further, serological studies utilizing virus-neutralizing tests have strengthened the concept of flavivirus cross-reactivity and segregated flaviviruses that are mosquito-borne, tick-borne, and those with no known arthropod vectors (5, 9). The antigenic similarities between flaviviruses are a secondary attribute that emerges owing to their genetic similarities. As a result, infection with one flavivirus results in both species-specific and flavivirus cross-reactive antibodies. The majority of flaviviruses that are relevant to human disease were organized into 8 serocomplexes plus 17 independent viruses that were not antigenically similar enough to warrant inclusion in a serocomplex (9) (Figure 1). Serocomplexes were defined by the ability of polyclonal post-immune sera against one flavivirus to neutralize others (10). Using DENV as an example, there are 4 serotypes of DENV (DENV1-4), which induce antibodies that are able to cross-neutralize each other to a certain degree, especially at high concentrations, in spite of those antibodies being insufficient to provide efficient neutralization and protection from secondary heterologous infections *in vivo* (10). In contrast, DENV-immune sera were unable to neutralize ZIKV, even though the serology indicated a relationship by another serological method [e.g. Enzyme-linked immunosorbent assay (ELISA)], supporting its close relationship to DENV but indicating that it falls into an independent serocomplex (11, 12). First described using human sera, these flavivirus cross-reactive immune responses appear to be consistent for multiple mammalian species, including rodents and non-human primates (13–15). During the acute phase of infection and disease, flavivirus cross-neutralizing antibodies can be induced, but these are usually not durable and cross-neutralization is not retained following a few months (12). Those exposed to multiple flaviviruses may also generate responses more difficult to decipher and which cross-neutralize viruses from distantly related serocomplexes (16, 17).

With the global spread of flavivirus vectors, increased human mobility, and increased vaccine coverage against flaviviruses, we are not only concerned with how pre-existing immunity could affect a heterologous challenge with a new virus from the same serocomplex (e.g. DENV2 infection followed by DENV1 infection) but also how immunity is influenced by sequential exposure to multiple flaviviruses from differing serocomplexes. Notwithstanding the potential of anti-flavivirus memory immune responses to influence subsequent infections, the high degree of immune cross-reactivity to flaviviruses makes infections, and prior exposures difficult to definitively identify when virologic confirmation is not possible (18).

## Functional Immune Outcomes of Sequential Flavivirus Infections

As humans have become more likely to experience more than one flavivirus infection during a lifetime, there is a need to understand how pre-existing immunity to a flavivirus impacts subsequent flavivirus infection outcomes. Early studies in humans exploring the nature of immune protection against flaviviruses observed that functional immune responses (virus neutralization) and the course of infection were modulated in the context of pre-existing flavivirus cross-reactive immunity (19, 20). While human studies have been largely correlation-based, studies using animal models have provided more definitive functional disease outcomes. For example, immunity to JEV and SLEV was protective against lethal WNV challenge in a hamster model (15). Similar cross-protection was observed when mice were immunized with Usutu virus and challenged with WNV (21). Within the same serocomplex, prior exposure to Kunjin or Murray Valley encephalitis (MVEV) viruses in pigs was also protective against JEV challenge (22). These studies suggest that cross-reactive immunity may be protective within the JEV serocomplex. In contrast, in a human DENV challenge study, immunity to DENV only provided lasting protection against a homologous DENV serotype (10, 19). Within the YFV complex, primary infection of rhesus macaques with Wesselsbron virus (from the YFV serocomplex) was protective against YFV challenge (13) and, more recently, examining the possibility of cross-protection against viruses in differing serocomplexes, JEV vaccination was shown to provide cross-protection against DENV and to increase the kinetics of the development of neutralizing antibody responses (14). Certain DENV or ZIKV-specific human monoclonal antibodies also can protect against a Spondweni virus challenge in immune compromised mice (23). However, primary infection with WNV (from the JEV serocomplex) or Banzi virus (from the YFV complex) failed to provide any protection against YFV challenge (13). The differences between cross-protection versus pathology have been more controversial in the context of how pre-existing immunity to DENV influences subsequent ZIKV infection. While some studies in immune compromised mice have suggested that DENV T cell immunity can be protective against ZIKV infection in adult mice (24), other studies in STAT2-KO mice, with impaired immunity, showed prior DENV immunity can enhance infection in adult mice and infection and fetal demise in pregnant mice (25, 26). In immune competent mice, pre-existing DENV immunity can enhance the development of a microcephaly phenotype in fetuses (27, 28), which is a key characteristic pathology of disease. These studies suggest that various factors determine cross-protection, some of which are discussed below.

## Flavivirus Cross-Reactive T Cell Responses

**FIGURE 2:**
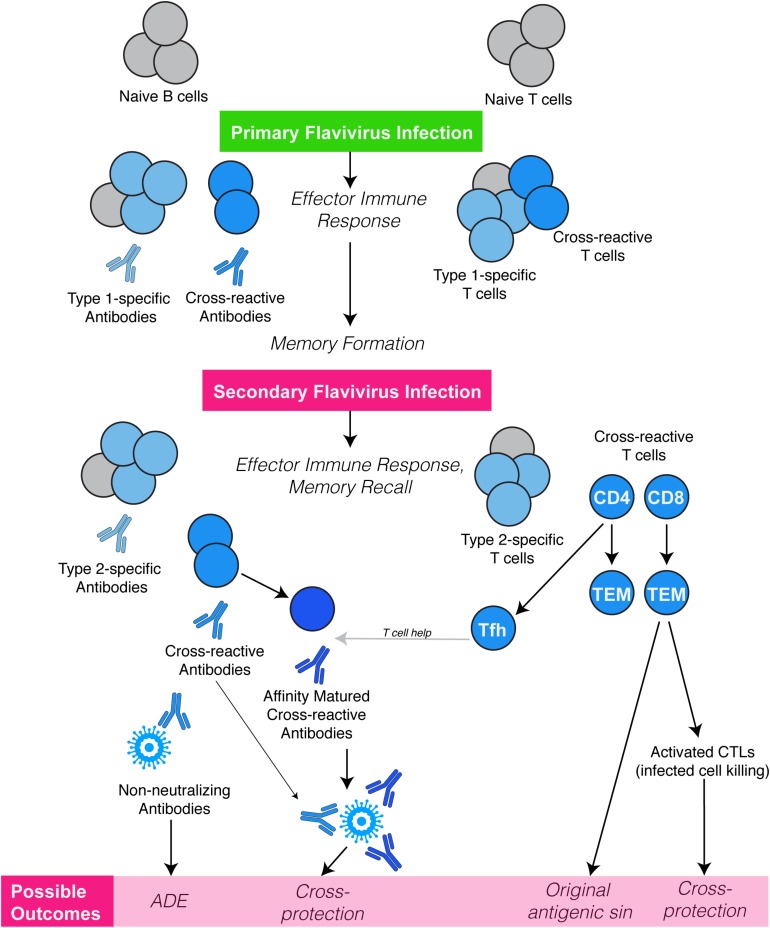
Flavivirus cross-reactive cellular immune responses. Infection or vaccination against a flavivirus (Type-1) results in a primary immune response dominated by generation of Type-specific antibodies and T cell responses, followed by a memory formation. Owing to their antigenic relatedness, flavivirus cross-reactive antibodies and T cells (CD4 & CD8) are also generated. A secondary challenge with a second flavivirus (Type-2) can potentially reactivate cross-reactive memory T cells, those, which have higher specificity for Type-1 than for Type-2 flavivirus. These weakly cross-reactive memory T cells may outcompete naive T cells that would be more specific for Type-2, resulting in T cell original antigenic sin. However, memory T cells could also provide cross-protection directly by acquiring CTL function resulting in enhanced killing of virus-infected cells. Importantly cross-reactive CD4 Tfh cells can be recalled in the lymph node germinal centers, providing help to B cells, and improving both affinity and avidity of antibodies that are cross-reactive and neutralizing. Flavivirus cross-reactive antibodies also interact in different ways during a secondary flavivirus infection. After primary infection, high affinity and Type-specific antibodies are produced, which can neutralize virus when present at optimal concentrations. However, during secondary heterologous flavivirus infection, pre-existing cross-reactive, sub-neutralizing antibodies may lead to opsonization of virus particles and enhanced uptake by various immune cells such as monocytes via Fc receptors, resulting in increased virus replication, a phenomenon termed antibody-dependent enhancement (ADE).

Various subsets of T cells are essential for efficient infection clearance and for the development of robust antibody responses against flaviviruses. Flavivirus reactive T cell epitopes have been identified in both viral structural and non-structural proteins and for CD8 and CD4 T cells (14, 29). Often, we expect that the T cell epitope must be 100% conserved to induce recall of a memory T cell from a previous infection; however, similar epitopes with only minor substitutions often are able to activate T cells as well although with potentially differing degrees of responses (30, 31). Early studies using murine T lymphocyte clones demonstrated T cell cross-reactivity similar to that observed using antibody neutralization tests (32, 33). For example, T cell clones specific to Kunjin or WNV showed cross-reactivity with MVEV or vice versa (32), suggesting T cell cross-reactivity within the JEV serocomplex. T cell cross-reactivity between two different serocomplexes has also been observed. CTL clones specific for DENV reacted and proliferated against Kunjin (JEV serocomplex), a virus from different serocomplex than DENV (34). Similarly, DENV or ZIKV induced CD8 T cell responses are also noted to be highly cross-reactive against each other (35). While the majority of flavivirus cross-reactive CD8 T cells are directed against viral non-structural proteins, studies using chimeric viruses have identified cross-reactive CD8 T cell epitopes that are present in the viral envelope protein (36). This suggests that both conserved non-structural proteins and variable structural proteins contribute toward the development of the flavivirus cross-reactive CD8 T cell repertoire. Interestingly, for human T cells, prior exposure to DENV skewed the immunodominance of ZIKV-specific T cells toward non-structural proteins (35), yet these cross-activated T cells retained their *ex vivo* functionality (37). This cross-reactivity may be beneficial for clearing a secondary infection since it has been shown that a vaccine utilizing non-structural epitopes of DENV can protect against DENV infection in mice (38). Still, the broad question of whether flavivirus cross-reactive CD8 T cells are protective or pathological in nature remains to be resolved. Indeed, within the DENV serocomplex, certain cross-reactive CD8 T cell epitopes have been associated with severe disease in humans (39, 40), illustrating the potential of cross-reactivity to reduce the efficiency of an antigen-specific response during a heterologous secondary challenge (10), a well-known phenomenon termed “original antigenic sin” (Figure 2).

CD4 T cells also have the potential to be cross-reactive and unique clones have been shown to have *ex vivo* stimulation in response to various flaviviral antigens (14, 41, 42). More recently, the functional role of serocomplex cross-reactive CD4 T cells has been identified, where they were shown to be associated with improved viral clearance during secondary serocomplex heterologous infection (14). Genetic similarity appeared to be a factor in cross-protection in this context, where JEV provided better CD4-dependent cross-protection against DENV than the more distantly related YFV in the mouse model (14). Importantly, CD4 T follicular helper (Tfh) cells with an effector memory phenotype were shown to be critical for efficient memory recall and in improving the quality of antibody responses, providing a mechanism of cross-protection (14). In humans vaccinated against JEV, cross-reactive T effector memory cells were also identified, which could be activated to proliferate and produce interferon-gamma in response to antigens from all of the heterologous viruses tested including DENV, YFV, and ZIKV (14). During heterologous DENV infections, CD4 effector memory T cells that have a T_EMRA_ phenotype, which is cytotoxic in nature, have also been identified (43, 44) and these may be present and recalled, in addition to cross-reactive Tfh cells, during heterologous flavivirus infection. It is probable that flavivirus cross-reactive CD4 T_EMRA_ cells are also present in individuals exposed to varied flavivirus infections or vaccines but their frequency may be very low. However, the antigen presenting molecule, MHC-II is highly polymorphic in humans and therefore flavivirus CD4 T cells responses can be restricted to certain HLA-types making identified cross-reactive epitopes likely relevant for only those with the given HLA-types (10). Virion structure also determines cross-reactive CD4 T cell immunodominance, as certain structural protein conformations can provide immunodominant epitopes that are conserved structurally but not genetically among flaviviruses (45, 46).

## Flavivirus Cross-Reactive Antibody Responses

The surface glycoprotein of a flavivirus consists of type-specific and serocomplex cross-reactive epitopes, owing to which the antibodies evoked are both type-specific and cross-reactive in nature (47–49). Structural determinants such as the conformation of epitopes and the presence of flavivirus conserved residues on the surface of the viral envelope (E) protein determine antibody binding and neutralization properties. For instance antigenic epitopes present on the surface of E protein, which consists of domains I, II, and III, are both linear and quaternary and immune activation to E generates antibodies that are largely neutralizing against the virus of same type (50–52). In the context of immune responses to E protein, antibodies that are neutralizing are better generated by the quaternary structures of E protein dimers, compared to the monomeric form of the protein (53). Moreover some E dimer epitopes are conserved across related flaviviruses and evoke antibodies that are cross-neutralizing to flaviviruses of differing serocomplexes, for example antibodies that can neutralize both DENV and ZIKV (54, 55). However, antibodies targeting the E protein fusion loop epitopes, which are conserved across various flaviviruses are also broadly cross-reactive but studies so far have indicated that they are poorly neutralizing (51). These weakly neutralizing cross-reactive antibodies may influence the course of subsequent flavivirus infection, as discussed below.

Both antibody specificity and concentration can govern the ability of antibodies to neutralize or enhance the uptake of virus by Fc-receptor bearing cells (56). In some cases, individual monoclonal antibodies that are capable of cross-neutralization may be identified from an otherwise sub-neutralizing pool (57). Conversely, weakly neutralizing antibodies often can lead to neutralization at high concentrations as was shown with WNV (58) which may factor in to the period of relative resistance to similar viral infections in the months immediately following infection (56). As a result of the decay of antibody in serum in the years following vaccination or natural infection, subsequent functional responses to new flaviviruses can be temporal (56).

There are antigenic relationships amongst flaviviruses that are rarely functionally tested *in vivo* in humans due to the infrequency of the two sequential infections. For example, antibody cross-reactivity to Yokose virus (from the Entebbe bat virus serocomplex) is also observed in patients’ sera infected or vaccinated with DENV or YFV (59). Yet, other sequential flavivirus challenges are much more common, such as the high probability of re-exposure to multiple serotypes of DENV or sequential exposures to DENV and ZIKV (60, 61). Much of our understanding of how flavivirus cross-reactive antibodies influence subsequent exposures to flaviviruses has been obtained in models of sequential DENV infections, where antibody-dependent enhancement of infection (ADE) by a heterologous serotype is consistently observed (10). This results from the binding of virus that is complexed with sub-neutralizing antibodies to the Fc receptors of immune cells including dendritic cells, monocyte/macrophages and mast cells (10). In this context, opsonization of virus can lead to enhanced infection in the immune cell types, resulting in both increased virus production and heightened production of pro-inflammatory mediators (10). Aside from opsonization, antibodies can also trigger alternate immune activation pathways such as Fc receptor cross-linking or antibody-dependent cellular cytotoxicity (ADCC) (10). During secondary heterologous exposures, mast cells activation is observed downstream of IgG or IgE cross-linking upon virus binding (62, 63). NK cell mediated ADCC also can trigger release of pro-inflammatory and cytotoxic mediators (64). ADE occurs not only for DENV, but for other combinations of viruses from differing serocomplexes, such as DENV and ZIKV (65). Indeed, ADE of the attenuated YFV strain 17D was shown to occur *in vivo* in humans, depending on JEV vaccine-induced antibodies from a prior immunization (66). However, Fc receptor activation and binding by virus-immune complexes may not always be detrimental to the host. For example, prior vaccination to JEV was shown to enhance uptake of YFV vaccine (strain 17D) by antigen presenting cells in mouse lymph nodes, leading to increased immunogenicity of the attenuated YFV vaccine (66) and suggesting a potential utility to ADE when it improves adaptive immune responses.

Phylogenetic analyses suggest a close relationship between DENV and ZIKV (67) and studies testing human anti-DENV sera demonstrated a high degree of cross-reactivity (11). As discussed, despite this high cross-reactivity, DENV-specific antibodies fail to cross-neutralize ZIKV (12), but may lead to opsonization of ZIKV viral particles (65). Not only was this shown to occur in conventional antigen presenting cells, dependent on the Fc gamma receptors, DENV antibodies can also enhance uptake of ZIKV in the syncytiotrophoblasts, and fetal endothelial cells of the placenta in a mechanism dependent on the fetal neonatal Fc Receptor, FcRN (27). In support of the role of antibody dependent enhancement of ZIKV pathology *in vivo* in humans, mothers with antibodies that were highly enhancing to ZIKV were shown to have fetuses (or children) with more severe microcephaly phenotypes (68). This pathological influence of DENV immunity during subsequent ZIKV infection may be specific for the context of pregnancy, since epidemiologic studies in humans suggest that ZIKV infection rates may be reduced in DENV immune non-pregnant individuals (69).

Pre-existing immunity also has the potential to improve cross-reactive antibody responses that are developed during a heterologous flavivirus infection. When JEV immunity led to faster induction of neutralizing antibodies to DENV, adoptive transfer studies showed that it was based on recall of a heterologous memory response and increased germinal center activity in lymph nodes, resulting in gains in antibody avidity and neutralization against DENV in JEV-exposed animals (14). However, there are also indications that the quality of antibodies can be impeded by prior flavivirus immunity. For example, in one study, individuals pre-vaccinated against YFV were shown to have a lower ratios of neutralizing to ELISA antibody titers (70), emphasizing that the quality of immune responses and not only the magnitude are important in determining the potential of cross-reactive immunity to induce protection versus pathology (Figure 2).

## Implications for Rational Flavivirus Vaccine Design

Herd immunity needs to be maintained to keep the population protected from certain flaviviruses for which vaccines are available. YFV is an example of this need since outbreaks of YFV in South America and Africa have occurred in recent years coinciding with reductions in vaccine coverage (71, 72). The success of the YFV vaccine has largely been attributed to its safety and effectiveness as an attenuated vaccine that induces immune activation of multiple pathways including innate responses, and effective T cell and antibody responses (73). A vaccine does not need to provide 100% of individual’s life-long durable protection to be effective, but the YFV vaccine often does. For this reason, it has been used as the “backbone” for other vaccines including the Sanofi Pasteur DENV vaccine, Dengvaxia (74). This means that the non-structural proteins of the 17D YFV vaccine were used to construct chimeric viruses with the structural proteins of each of the DENV1-4 viruses (74). Aside from its validated safety, the YFV backbone was also chosen for this DENV vaccination strategy because the replication rates could be closely matched for all serotypes, promoting similar antigen persistence *in vivo* (74). This was to counteract the problem that was discovered early on in DENV vaccine design, where one or two of the 4 serotypes replicated much more efficiently *in vivo*, leading to immune dominance and poor coverage against multiple serotypes (75). Dengvaxia (76) has been licensed in several countries and although the effectiveness differs between serotypes it is not clear if this is due to residual differences in antigen persistence between chimeric vaccine strains or due to the influence of prior DENV immunity present in the various populations where the vaccine was tested. More pressing, in spite of the moderate and acceptable levels of efficacy shown, some safety concerns were raised, including that children, which were likely a surrogate for flavivirus-naïve individuals since the vaccine was tested in hyperendemic regions, were more likely to require hospitalization (77). Since then, it has been surmised that an ADE-like response may be occurring in certain vaccinated individuals having breakthrough cases, which is induced by the antibodies to the vaccine (78). It is also possible that the mismatch of the virus non-structural proteins to DENV is a contributing factor to the development of breakthrough cases in vaccinated individuals (10). Supporting this, T cell responses in YFV vaccinated mice were not efficiently recalled in DENV stimulated T cells and YFV-induced CD4 T cell responses showed little cross-reactivity for DENV epitopes (14). Human cross-reactive T cell responses between YFV and DENV have been detected (14). Alternate backbones have been used for DENV vaccine strategies, including the JEV backbone, which was protective in mouse models (79) and a conserved DENV backbone, itself (80). The Takeda vaccine, for example adopted this strategy, using a DENV2 backbone with DENV1-4 structural proteins (80, 81). This strategy has the potential to provide increased recall of vaccine-induced T cell responses during natural infection with DENV viruses. Recently a clinical trial demonstrated efficacy of the Takeda vaccine in humans in a dengue-endemic region as well (82), although the long-term effects of vaccination and the persistence of protection will need to be monitored alongside the potential of any breakthrough severe disease. Importantly these vaccines illustrate how homologous and heterologous immunity to flaviviruses can change the protective capacity of a vaccine and potentially even influence its safety. Although we are concerned with the potential of cross-reactive immunity to induce pathologies following vaccination against flaviviruses, the indications that cross-protective immunity can be induced also highlight the potential of rationale design of cross-protective vaccines that are effective against multiple flaviviruses in the future.

## Outlook

In spite of many recent advances toward flavivirus directed vaccines, the world wide burden of flaviviruses is actually increasing. The potential of cross-reactive immunity to influence the infection outcomes and the fact that the immune profile of individuals can change over time, being different in the months, versus years, versus decades following infection, emphasizes the need to continue studying how cross-reactive immunity works and influences infection outcomes. Compounding this is the issue of flavivirus emergence. The flavivirus genus contains diverse viruses that are present in the environment and hidden in unexplored reservoirs. The recent emergence of WNV in North America in 1999 (83), ZIKV in the South Pacific in 2013-2014 (84) and South America in 2015-16 (61) and resurgence of YFV (72), in spite of a highly effective vaccine, and growing vaccine coverage for DENV emphasizes the need to consider how cross-reactive immunity will influence infection by those flaviviruses we expect and also those that don’t have a significant burden in humans at this time.

## Author Contributions

AS and AR conducted literature search and analysis, and designed and wrote the manuscript.

## Conflict of Interest

The authors declare that the research was conducted in the absence of any commercial or financial relationships that could be construed as a potential conflict of interest.
